# What is the reward? Medical students’ learning and personal development during a research project course

**DOI:** 10.3402/meo.v20.28441

**Published:** 2015-09-04

**Authors:** Riitta Möller, Maria Shoshan, Kristiina Heikkilä

**Affiliations:** 1 Department of Medical Epidemiology and Biostatistics, Karolinska Institutet, Stockholm, Sweden; 2 Department of Oncology-Pathology, Karolinska Institutet, Stockholm, Sweden; 3 Department of Health and Caring Sciences, Faculty of Health and Life Sciences, Linnaeus University, Kalmar, Sweden

**Keywords:** learning outcomes, qualitative research, medical students, science and scientific, learning to research

## Abstract

**Background:**

Until recently, the outcome of medical students’ research projects has mainly been assessed in terms of scientific publications, whereas other results important for students’ development have been less studied. The aim of this study was to investigate medical students’ experiences of learning as an outcome of the research project course.

**Method:**

Written reflections of 50 students were analyzed by manifest inductive content analysis.

**Results:**

Three categories emerged: ‘thinking as a scientist’, ‘working as a scientist’, and ‘personal development’. Students became more aware about the nature of knowledge, how to generate new knowledge, and developed skills in scientific thinking and critical appraisal. Unexpectedly, effects on personal characteristics, such as self-confidence, self-discipline, independence, and time management skills were also acknowledged.

**Conclusions:**

We conclude that individual research projects enhance research-specific skills and competencies needed in evidence-based clinical work and are beneficial for personal and professional development.

All doctors must understand the scientific process and must in their future practice be able to evaluate and integrate new knowledge (1). Individual research projects are one way to develop these skills, and they are taking on a more central role in higher education, especially in medicine (2).

Academic outcomes, for example, publications and presentations, have been measures of success of research project courses in medical education. Reports from three US medical schools show that 40–75% of the students had published at least one paper after their research period, and about half had given a presentation at an extramural meeting (3, 4). Similar results have been reported by others and are presented in the most complete synthesis to date by Bierer and Chen (2). However, publications in medical science are usually the result of teamwork and do not necessarily reflect the value of the research project course as a learning opportunity for the student.

By comparison, what medical students learn during research projects has been less studied. Several publications present only a description of the course, or experiences of the project administrators, albeit sometimes with students’ feedback (1, 4–8). Studies on students’ learning have often used questionnaires with scales and/or closed-end questions that limit respondents to a list of answers. Houlden et al. (9) surveyed 60 medical students after a mandatory 8-week ‘Critical Enquiry’ course and found that half of the students experienced increased confidence in performing literature searches, critical appraisal, and manuscript preparation. Burgoyne and colleagues (10) surveyed 317 medical students of whom 21% had taken or were currently taking a research-focused course, or had for a previous degree undergone a research program. The latter possessed significantly higher research-specific skills than the school-level entrants. As the study did not differ between students at different stages in their education, it is difficult to draw stringent conclusions about the learning benefits. A deeper analysis (11, 12) based on interviews of 76 students from four colleges after a 10-week summer research program in, for example, physics, chemistry, or biology, showed increased confidence and gains in scientific knowledge and practical skills.

Experiential learning is referred to as learning through real-life experience emphasizing social interaction (13, 14). Vygotsky described the zone of proximal development that represents the gap between the learner's developmental level in a certain task (what the learner can do without help) and the level that can be achieved with guidance by the ‘more knowledgeable other’ (e.g., supervisor) (13). The core idea is that, with assistance, students are able to complete tasks that they are not able to do on their own (15).

Another sociocultural theory of experiential learning focuses on communities of practice (COP) that are groups or networks in which the members, based on a common interest in a specific area, share knowledge and problems, and discuss and learn from each other (16). Thus, students who do research projects in authentic COPs have access to expert knowledge, cooperation, and support, and thereby the opportunity to develop professionally and personally (12, 17).

In summary, there is a lack of insight into student-defined learning as an outcome of longer research projects that are finalized with a formal, single-author scientific report. Such courses have become mandatory at medical as well as other university programs in Europe after implementation of the Bologna process. The aim of the current study was to investigate medical students’ experiences of learning and other outcomes of individual, advanced-level research projects, based on students’ written reflections after the course.

## Methodology

### Study setting

The educational context of this study is a medical university (Karolinska Institutet) with a 5.5-year long medical program. The program introduced a new curriculum in 2007. The first 2 years cover basic sciences (e.g., cell biology, anatomy, and physiology) and the last 3.5 years mainly clinical education (e.g., medicine, surgery, and pediatrics) including a total of 23 weeks of electives during the clinical courses. Three so-called threads (professionalism, primary care, and scientific education) run throughout the program. The mandatory course ‘Degree project in medicine’ (20 weeks; 30 European Credit Transfer System (ECTS) credits) was introduced in 2010 and placed in term 7. It constitutes the largest individual and most science-directed project that the students carry out, and was therefore considered a suitable context for this study. The overall aim of the course is to provide students with deeper understanding of performing and evaluating medical research. They individually, but under supervision, plan and carry out a research project and present a formal research report. The aim is not to train the students to become researchers or to write a paper for publication, but to bring them to understand the scientific basis of medicine and interpret scientific findings in order to become clinicians who are scientifically skilled. The scope of the final report is about 20–35 pages and the structure is similar to that of scientific papers. The target group is fellow students and other readers of scientific medical literature with basic medical education. The projects involve authentic empirical research in basic, clinical, or translational science, usually within a research group. The projects are supervised by an active researcher in the topic area and with at least a PhD degree. The first author of this paper was the course director for the course at the time of the study.

There are two mandatory checkpoints: seminars for presenting an initial project plan within the first 2–3 weeks and a midterm report. This creates two occasions for students to practice oral presentations and critical appraisal of other students’ presentations and reports. Another purpose is to jump start the writing process and for teachers to monitor the suitability and progression of the projects. The project plan includes a short introduction, aims, an overview of material and methods, and a few references. The midterm report consists of a preliminary version of the final report's introduction, aims, material and methods, and, if possible, some results, and a progress report on, for example, how far data collection and analysis have advanced as well as a time plan for the rest of the course. Both the project plan and midterm report are presented and peer-reviewed at separate seminars. The final examination comprises the complete written report, an oral presentation and critical appraisal of another student's report, and also a written reflection on personal experiences of learning and development during the course.

### Study design

A qualitative approach was chosen for this study based on students’ written reflections of personal experiences of learning and development during the course. The theoretical starting point was the epistemological assumption that knowledge and understanding are socially constructed (18). The current findings were the result of analysis of these reflections. Rather than reflecting an objective truth, the findings aim to contribute to the understanding of a complex phenomenon and to be reasonable and generalizable interpretations (18, 19).

### Material and sampling

Writing assignments that promote reflective capacity are becoming more common in medical schools. Dewey has claimed that reflection on experience belongs to the very heart of education, because education is basically the reconstruction or reorganization of experience (20, 21). Many agree that reflection is associated with self-awareness and adult learning (22–24). Thus, written reflections are well-suited for the analysis of learning experiences.

In 2012, when data were collected, 122 of the 143 medical students turned in written reflections of 3–4 pages and based on three prompts: what the student learned during the course, the significance of collaboration, and what the student will take with himself/herself for the future. Responses to the first and third prompts were analyzed.

To obtain data, all 122 reflections were first read through by the first author, and texts rich in information and showing variation in learning experiences were selected (25, 26). Initially, 20 content-rich reflections were selected for analysis, whereafter more were added until no new aspects emerged from the material (i.e., saturation was reached). In total, 50 reflections from students aged 24–48 were analyzed. Of these reflections, 45 were written in Swedish and 5 in English.

### Data analysis

All the reflections were anonymized, and then numbered 1 to 50. Each reflection was analyzed separately. The anonymized reflections were the units of analysis and comprised 154 pages of text. The analysis was conducted using inductive manifest content analysis based on an iterative, constant comparison coding technique (27, 28). Codes, subcategories, and categories were developed from the text without any predetermined coding scheme.

To obtain a sense of the whole, the reflections were first read through several times and then entered into the NVivo software package (29) for coding. Then, meaning units were identified (27). A ‘meaning unit’ was a statement that included one piece of information of interest for the aim of the study: it could be less than a sentence but never more than five sentences. The meaning units were read through several times and labeled with a code, and codes were grouped into subcategories according to their content. Finally, three main categories (Table 1) were formulated to capture the manifest content. The study was approved by the Regional Ethical Review Board Karolinska Institutet, Stockholm, Sweden (Dnr 2010/1100-31/1 and Dnr 2011/493-32).

**Table 1:** Overview of subcategories and categories generated from students’ reflections

Subcategories	Category
The nature of scientific knowledge	Thinking as a scientist
Attaining a scientific attitude	
Scientific research in practice	
Scientific information competence	Working as a scientist
Scientific communication	
Self-confidence	
Independence and collaboration	
Time management	Personal development
Self-discovery	
Career plans	

## Results

This analysis of students’ reflections, regarding learning during the research project course, resulted in three main categories. These were: thinking as a scientist, working as a scientist, and personal development (Table 1).

### Thinking as a scientist

The category thinking as a scientist is based on two subcategories that emerged from the reflections: the nature of scientific knowledge and attaining a scientific attitude.

#### The nature of scientific knowledge

Conducting a research project prompted students to reflect on the nature of knowledge. The epistemological perspective seemed to change to one that was more relative and complex than the students’ initial perspective. Students described how they came to realize that there is no absolute truth, and that today's knowledge is not necessarily the knowledge of tomorrow. One student commented:

Something I will take with me in the future is the realization that the information we have today, and on which our practices are based, need not be the absolute truth. (Student 8)

The students noted that one must be prepared to revise one's point of view and be critical. Moreover, the magnitude of the collected body of knowledge and the obvious effort that is needed to generate knowledge seemed to come as a surprise:

Another important insight for me has been how much painstaking work and research lies behind a few lines in a report, hours to months or years of work may lie behind a sentence in a scientific report. (Student 10)

#### Attaining a scientific attitude

A scientific attitude was found to entail critical thinking based on logical reasoning, inquisitiveness, and factual knowledge. The students realized that critical thinking and scientific attitude takes time to learn but can be developed by regular practice. The scientific attitude was seen to develop as a result of reading papers, critical appraisal of scientific texts, data collection, and scientific writing.

By combining my logical reasoning with my medical knowledge and inquiry as a starting point when reviewing recent literature, I ended up using critical thinking skills automatically. What I also discovered was that this is a mindset that can be developed just like any other skill; Like any other skill you must practice it regularly. (Student 35)

As a consequence, students developed open-mindedness, which in turn modified how they worked. They learned to question and view things from several perspectives, and to better distinguish between the relevant and the nonrelevant. This development also facilitated interpretation and evaluation of scientific studies. Students noted that published interpretations and conclusions can often be questioned, and that scientific thinking is of value in clinical work. The development of a scientific attitude was consequently considered an important outcome of the course.

… Serious critical thinking should not only be practiced by researchers …. I also became convinced about the importance of using it in clinical practice in the future, where the ability to discern truth from modified truth is valuable. (Student 35)

### Working as a scientist

This category was based on three subcategories: scientific research in practice, scientific information competency, and scientific communication.

#### Scientific research in practice

The core of this subcategory is the students’ descriptions of the practical research process, about which they knew little before the course. Previous teaching in science had been theoretical and had included only short practical exercises, whereas the research project course allowed students to learn the craft, from planning to writing a report in an authentic environment.

The main thing I have learned, I believe, is the actual realization of how the scientific process works and how to initiate, plan and implement a project of this size in practice. (Student 43)

Students wrote that they had gained not only subject knowledge but also knowledge about research ethics, laboratory techniques, and other methods. Their ability to analyze data and their competence in statistics had improved. Consequently, several students found research less frightening and demanding after taking the course. Moreover, students noticed that although research projects were planned and prepared beforehand, unexpected events often occurred, which led to the insight that scientific research is not like following a recipe. Instead, many activities and plans are revised, based on experience from previous mistakes. Problem solving was found to be key in a research process.

Solving problems, and looking at a problem in a new way, solving it, finding new ways, trying them, failing, continuing and trying a new way, failing again, trying something new that might succeed … this is the heart of research. (Student 38)

Many students came to realize that scientific research entails constant collaboration. They described how inspiring, knowledgeable, supportive, and motivating a research team can be, and how much they had learned from being a part of a scientific community.

The image ordinary people probably have is that of a lonely researcher who works hard for himself in a half-dark lab in the evenings, finally crying ‘Eureka!’ not surrounded by anyone …. This picture could not be more wrong. Research requires collaboration ALL the time. (Student 32)

The drawbacks of being a scientist were also noticed and some students made negative statements about the research environment. They found the working climate to be competitive and cynical. Some students described scientists’ work as demanding, time-consuming, and repetitive.

I have had many discussions during the semester with various scientists about their views on the world of research and realized that it is largely a cynical world. (Student 30)

#### Scientific information competency

It appeared that literature searches progressed from substandard or routine searches to more focused and effective ones. Students learned to build and use search strategies in different databases wherefore their ability to manage information overload improved. Experiencing an improved ability to find relevant information also increased the desire to seek new information.

It is easier for me to use all the databases that are available. I feel that I can better manage the abundance of available information and select information for a specific purpose. (Student 6)

The students’ approach to scientific literature changed during the course. Reading scientific papers was not new to them, but they had never had to read so much scientific literature in such a short time period. Reading was first considered difficult, but most students developed techniques of skimming through papers, to then decide whether or not to read them thoroughly. Consequently, reading scientific literature became a more natural way of acquiring information.

I have previously felt scientific papers in English were complicated and difficult to understand, so I often neglected to read them. During this semester, I have really put my ‘fear’ of scientific papers to the test and worked out a technique to read them. Now it feels perfectly natural to seek current knowledge in scientific articles. (Student 4)

Students frequently mentioned that they had learned or further developed their critical appraisal skills, in particular by reading scientific papers, and by writing the project report. Critical appraisal was deemed useful for clinical practice, and many of the students considered this to be the most significant outcome.

Critical review of scientific texts is perhaps the most important thing I learned and what I will use the most in everyday clinical practice in the future. (Student 20)

#### Scientific communication

Scientific writing – the ability to write clearly, objectively, concisely, and without emotional expressions – was considered difficult and time-consuming. Students stated that it was like learning a new language: the more one practices, the easier it gets. They also discovered that the more they read, the easier it became to write. In addition to reading and analyzing the language in the papers, writing skills were developed by discussions with supervisors and classmates, oral presentations, peer review for seminars, and continuous feedback. The students gradually learned that scientific writing is a process.

Over time, I learned that you go back and change all the time. That writing is a process that takes time. You change your text. You make it better. In the beginning, I tried to imitate the way language is used in the articles, but towards the end I stopped checking. I had begun to think in a different way, and therefore also write in a different way. (Student 13)

Students gave several oral presentations during the course, which developed their presentation skills. Doing this during the course was different from earlier presentations, as they now owned the presentations and their content, rather than having been assigned a certain topic.

It was very different from previous presentations, which usually have involved a certain topic or a disease. Now it was instead one's own work with own questions and reflections and own results and interpretation of these. It was exciting and at the same time a little challenging …. (Student 50)

The development of presentation skills was connected to subsequent scientific discussions. That students listened to each other's presentations, and participated in discussions, helped them to continue honing their own work.

To listen to others who present their projects, to discuss, to receive criticism, and to defend one's own work has been developing. It has also helped me to progress in my project by bringing up new thoughts and ideas. (Student 37)

Students wrote that their ability to discuss science, not only the project itself but also scientific publications, improved. Although of different characters, discussions with supervisors, other researchers, classmates, and family members were all important in the learning process. In different ways and at different levels, the discussions led to a broader and deeper understanding of the research topic and developed the communication skills.

By discussing my work with my friends and family, or rather telling them about it, I have deepened my own understanding of the subject, become better at communicating in an understandable way, and developed the ability to pay attention to what might be difficult to understand for those not familiar with the subject. (Student 25)

### Personal development

The most salient learning outcomes and benefits were related to personal development. This category included five subcategories: self-confidence, independence and collaboration, time management, self-discovery, and career planning.

#### Self-confidence

Students gained self-confidence not only regarding their ability to do scientific research but also their future work as a physician. Increased self-confidence was related to successful experiments or other results, to the ability to solve problems, and to the overall ability to carry out a project.

During my time in the lab I developed good self-confidence in terms of my ability to pursue research. I feel today more convinced that I possess the combination of knowledge, skills, and curiosity that are necessary to conduct research. (Student 27)

#### Independence and collaboration

Students learned to work independently and experienced that they had to take responsibility for the whole project. The development of independence was described as a process. It was initially distressingly difficult to know what to do and how to proceed, but the subsequent process toward independence enhanced the students’ motivation and ownership of the project. Altogether, this was considered valuable for their future professional work.

I have learned that I have the sole responsibility for what I do. I think this is also important for the future work with the patients. It is my responsibility to know what to do for my patients. (Student 5)

Collaboration was found to provide new perspectives, resulting in discussions, sharing of new ideas, and feedback from persons other than supervisors. Moreover, collaboration also prevented mistakes. Students greatly appreciated these learning opportunities and knowledge-sharing communities.

I have learned what a positive environment a lab can be. The eagerness of my fellow labworkers to help me has rescued me from many tricky situations I think such environments, in which everyone shares their knowledge and helps each other, are important for the scientific community and benefit everyone. (Student 33)

On the other hand, students found it challenging to balance independence and collaboration. They wrote that it is important to listen to those who are more experienced while it is at the same time necessary to try to develop independence and self-confidence.

#### Time management

Time management skills regarding activities related to research, and students’ personal life, improved. These skills included ability to allocate time; set goals; and organize, schedule, and prioritize activities, both on a daily and on a long-term basis. An important discovery was that things take longer time than expected. Students described how they also had to learn to take into account their supervisors’ schedules. Overall, students felt that time management skills were worth developing for their future profession.

This term has brought planning to a new level …. I created my own schedule in order to finish my report. This schedule included meetings with supervisors and other key persons … with limited timeframes, so I also had to develop the ability to use time during these meetings in a very efficient manner. (Student 37)

#### Self-discovery

During the semester, I also learned about myself: I aminterested in people, and want to help people andconnect with other people. This has reduced mydoubts about medicine being the right choice for me.(Student 24)

Some students emphasized elements of self-discovery, both in a positive and a negative way. Their self-discipline and initiative were enhanced. Some students found that because the project work required considerably more independence than previous courses they had developed more efficient working methods. Others realized they needed coworkers, that is, they did not like to work alone. Some become aware of personality traits that might influence their personal life and their future as researchers.

The traits essential for a successful scientist are perseverance and patience, two qualities that do not necessarily define me. (Student 49)

An important outcome for some students was that they developed the courage to ask questions and to handle obstacles; asking questions and advice was no longer considered a weakness. On the contrary, it proved to be an effective way of learning. Indeed, students learned that mistakes and obstacles are an intrinsic part of the research process. This was eloquently described by one student:

I have also, with the help of my supervisor, learned to handle what might have been the hardest part of the project work, to fail. At first it was very difficult for me …. I have now learned that failed experiments are common when doing basic research. (Student 27)

#### Career planning

Although research as an option for the students’ future profession had been unclear before the course, almost all students wrote that their career plans now included a wish to do research. The most common notion was to become a part-time clinical researcher; the possibility to combine clinical work with part-time research was a positive surprise and opened up for new career plans.

When I started medical school, it was in some way the researcher's role I was curious about, but I quickly pushed it aside after understanding that my interest mainly lies in the clinical work. Now, however, I realized that the one does not exclude the other. (Student 18)

The motivation after the course to conduct research was the desire to contribute to the development of medical knowledge and of health care, both of these elements being an essential part of every physician's work. Personal benefit and development were other reasons. Some students also wished to teach in the future and described how their supervisors had been role models for them.

I also became interested in teaching. Thanks to my supervisors who have shown that it is possible to work clinically, do research and teach, I also became interested in combining all these three parts. (Student 40)

Working as a scientist was not, however, the kind of career all students wanted. Negative statements were made about the requirements for the research project report, problems with writing, and the lack of an expected improvement in a specific skill such as statistics. In addition, some students were reluctant to pursue research because they preferred interaction with the patients.

The stimulus it provides to listen to people's life stories, help them with their ailments, and diagnose diseases is undoubtedly superior to working as a junior researcher – even in a top-down, flow-fixed health care organization. (Student 45)

## Discussion

This study is one of the few studies that focus on outcomes, in terms of learning, of the increasingly common one-term mandatory research project courses for medical students. Rather than assessing the number of resulting publications, which has been done in several previous publications (2, 30), the study reports outcomes as revealed by qualitative analysis of students’ own written reflections. Importantly, our results thus relate to the students’ own perspective and indicate corollaries that may be of consequence for their future profession. The reflections show that research-related skills developed in alignment with the intended learning outcomes. A major finding was, perhaps unexpectedly, that the students placed such emphasis on the impact on personal development.

In the past, basic professional education was sufficient for a lifetime career. Our results indicate that the research project course helped students realize that today's physicians need to remain scientifically updated and that scientific knowledge is dynamic, not static. As a course outcome, the emergence of such an understanding has not often been reported in the literature, but is consistent with a study on college students by Seymour et al. (11). Along with improved confidence in, for example, critical appraisal of scientific information, this understanding is a prerequisite for intellectual progress and scientific attitude.

We place our findings within the framework of experiential learning (13, 14) and the notion that knowledge and understanding are socially constructed (18). The present results support and illustrate the educational functions and benefits of acting within a COP (16). We find it significant that although not asked to, the students described their learning processes, for example, how discussing science with people within as well as outside the research team supported development of communication skills as well as personal scientific understanding. We noted that this was experienced within quite different COPs (data not shown).

Our findings indicate other generic skills and insights as outcomes of the course, notably development of independence, in terms of responsibility both for the project and for own learning needs, time management, and increased self-knowledge. These findings along with the above aspects of personal development were not the result of theoretical studies but important steps toward a structured and rational mindset that were conveyed and nurtured by social interactions in a research context (13–15). We therefore encourage all attempts to increase the awareness among supervisors of their crucial roles as role models and communicators.

However, none of the students indicated a connection between improved communication skills and clinical work, for example, communication with patients or other health care workers. Similarly, neither increased teamwork skills nor the importance of teamwork in science were associated with the future professional work. These findings are new though not very encouraging. They may, however, depend on the prompts; it would be interesting to investigate responses to prompts that more specifically address these issues, in particular as communication and teamwork skills in clinical environments are related to increased patient safety and health care efficiency (31). We therefore suggest that practicing teamwork and communication during research projects should be more explicit learning outcomes, which would further strengthen the link between scholarly work and clinical education.

Research training during medical schools has been suggested to increase interest in research careers (32, 33). Quantitative studies have examined this by reporting percentages of students aiming for research careers (2). Our study, in concordance with a few others (34, 10), shows that as students become more aware of research and its relevance for medicine they also start involving research in their career plans. Surprisingly, many students had been unaware that engaging in research does not exclude clinical work but exposure to research is likely not enough to establish it as career choice. Based on social cognitive theory, Bakken and colleagues (35) suggested that person–environment interactions as well as outcome expectations shape learning experiences and career choices. Thus, supervisors as role models and students’ capability to cope with uncertainty, stress, and anxiety during the research project may, among other factors, impact career choices. These aspects have not been investigated in the current study. We believe, however, that medical students constitute an important source of future researchers wherefore research projects courses may well encourage scientific careers.

Mandatory research training in medical education is sometimes criticized for usurping time and effort in an already overloaded curriculum (34). In addition, students’ research projects often appear as a final-year assessment when students are expected to show a mastery of their discipline. Our findings indicate long-term personal and professional benefits of a research project course and support placing it earlier in the education; this enables students to implement their experiences during the final clinical education, and to further appreciate the value of social interaction in a good COP (14, 15). In addition, as many students came to realize the need to question and evaluate information and tradition, the course may enhance the understanding of the connection between optimal patient care and evidence-based medicine; this is another interesting subject for further investigation.

Several limitations warrant a discussion. First, credibility (19, 27) may be questioned in that students’ descriptions are subjective, and may be anywhere on a scale between ‘genuine’ and ‘dishonest’ (36). Most students wrote their reflection during the examination period, which may have caused stress and thereby immoderate feelings. However, both positive and negative aspects were revealed, indicating that students felt comfortable enough to express their true reflections. Moreover, the reflections were anonymous. In order to add credibility, we used a comprehensive sampling providing a variety of participants and breadth in the data. Investigator triangulation was used engaging all authors in the analysis. The coding and the definition of categories have been discussed and checked by all the authors. To attain dependability, the data were collected until no new categories emerged (saturation) and were continually re-examined using the insights that emerged during analysis. Finally, this study has been conducted at one university wherefore the context has been described at some length (27). This enables the reader to evaluate transferability to other settings.

Altogether, the results illustrate that individual research projects may be beneficial for both personal and professional development. Future studies should investigate supervisors’ perceptions of students’ learning, supervisor–student relationships, and their impact on learning, and examine the roles of the different learning environments at hand and the impact of research project courses on the numbers of MDs and PhDs.

## References

[1] Smith FG, Harasym PH, Mandin H, Lorscheider FL (2001). Development and evaluation of a research project program for medical students at the University of Calgary Faculty of Medicine. Acad Med.

[2] Bierer B, Chen HC (2010). How to measure success: the impact of scholarly concentrations on students – a literature review. Acad Med.

[3] Dyrbye LN, Davidson LW, Cook DA (2008). Publications and presentations resulting from required research by students at Mayo Medical School, 1976–2003. Acad Med.

[4] Laskowitz DT, Drucker RP, Parsonnet J, Cross PC, Gesundheit N (2010). Engaging students in dedicated research and scholarship during medical school: the long-term experiences at Duke and Stanford. Acad Med.

[5] Rosenblatt RA, Desnick L, Corrigan C, Keerbs A (2006). The evolution of a required research program for medical students at the University of Washington School of Medicine. Acad Med.

[6] Gotterer GS, O'Day D, Miller BM (2010). The Emphasis program: a scholarly concentrations program at Vanderbilt University School of Medicine. Acad Med.

[7] Boninger M, Troen P, Green E, Borkan J, Lance-Jones C, Humphrey A (2010). Implementation of a longitudinal mentored scholarly project: an approach at two medical schools. Acad Med.

[8] Green EP, Borkan JM, Pross SH, Adler SR, Nothnagle M, Parsonnet J (2010). Encouraging scholarship: medical school programs to promote student inquiry beyond the traditional medical curriculum. Acad Med.

[9] Houlden RE, Raja JB, Collier CP, Clark AF, Waugh JM (2004). Medical students’ perceptions of an undergraduate research elective. Med Teach.

[10] Burgoyne LN, Flynn SO, Boylan GB (2010). Undergraduate medical research: the student perspective. Med Educ Online.

[11] Seymour E, Hunter AB, Laursen SL, Deantoni T (2004). Establishing the benefits of research experiences for undergraduates in the sciences: first findings from a three-year study. Sci Educ.

[12] Hunter AB, Laursen SL, Seymour E (2007). Becoming a scientist: the role of undergraduate research in students’ cognitive, personal, and professional development. Sci Educ.

[13] Vygotsky LS (1978). Mind and society: the development of higher mental processes.

[14] Yardley S, Teunissen PW, Dornan T (2012). Experiential learning: AMEE guide No. 63. Med Teach.

[15] Wass R, Golding C (2014). Sharpening a tool for teaching: the zone of proximal development. Teach High Educ.

[16] Wenger E (2000). Communities of practice and social learning systems. Organization.

[17] John J, Creighton J (2011). Researcher development: the impact of undergraduate research opportunity programmes on students in the UK. Stud High Educ.

[18] Illing J, Swanwick T (2010). Thinking about research: frameworks, ethics and scholarship. Understanding medical education. Evidence, theory and practice.

[19] Guba E, Lincoln Y, Denzin N, Lincoln Y (1994). Competing paradigms in qualitative research. The handbook of qualatative research.

[20] Dewey J (1933). How we think.

[21] Wear D, Zarconi J, Garden R, Jones T (2012). Reflection in/and writing: pedagogy and practice in medical education. Acad Med.

[22] Mezirow J (1991). Transformative dimensions of adult learning.

[23] Mann K, Gordon J, MacLeod A (2009). Reflection and reflective practice in health professions education: a systematic review. Adv Health Sci Educ.

[24] Sandars J (2009). The use of reflection in medical education: AMEE Guide No. 44. Med Teach.

[25] Sandelowski M (1995). Focus on qualitative methods. Sample size in qualitative research. Res Nurs Health.

[26] Creswell JW (2007). Qualitative inquiry & research design. Choosing among five approaches.

[27] Graneheim UH, Lundman B (2004). Qualitative content analysis in nursing research: concepts, procedures and measures to achieve trustworthiness. Nurs Educ Today.

[28] Elo S, Kyngäs H (2008). The qualitative content analysis process. J Adv Nurs.

[29] Bazeley P (2007). Qualitative data analysis with Nvivo.

[30] Van Eyk HJ, Hooiveld HW, Van Leeuwen TN, Van der Wurff BL, de Craen JM, Dekker FW (2010). Scientific output of Dutch medical students. Med Teach.

[31] CAIPE (1997 and 2006) Interprofessional education – a definition.

[32] Segal S, Lloyd T, Houts PS, Stillman PL, Jungas RL, Greer RB (1990). The association between students’ research involvement in medical school and their postgraduate medical activities. Acad Med.

[33] Lopatto D (2004). Survey of Undergraduate Research Experiences (SURE): first findings. Cell Biol Educ.

[34] Laidlaw A, Aiton J, Struthers J, Guild S (2012). Developing research skills in medical students: AMEE Guide No. 68. Med Teach.

[35] Bakken LL, Byars-Winston A, Wang MF (2006). Viewing clinical research career development through the lens of social cognitive career theory. Adv Health Sci Educ.

[36] Maloney S, Tai JH, Lo K, Molloy E, Ilic D (2013). Honesty in critically reflective essays: an analysis of student practice. Adv Health Sci Educ.

